# Characterization of IL-10-producing neutrophils in cattle infected with *Ostertagia ostertagi*

**DOI:** 10.1038/s41598-019-56824-x

**Published:** 2019-12-30

**Authors:** Lei Li, Hongbin Si, Shu-Wei Wu, Jonatan Orangel Mendez, Dante Zarlenga, Wenbin Tuo, Zhengguo Xiao

**Affiliations:** 1 Department of Avian and Animal Sciences, University of Maryland, College Park, MD 20742 USA; 2 Animal Parasitic Diseases Laboratory, USDA/ARS, Beltsville, MD 20705 USA

**Keywords:** Parasitic infection, Parasite immune evasion

## Abstract

IL-10 is a master regulator of immune responses, but its cellular source and function in cattle during the initial phase of immune priming have not been well established. Despite a massive B cell response in the abomasal draining lymph nodes in *Ostertagia ostertagi* (OO)-infected cattle, protective immunity is slow to develop, and partial protection requires years of repeated exposure. In addressing this problem, our initial hypothesis was that B cells produce IL-10 that downregulates the host protective immune response. However, our results showed that neutrophils made up the majority of IL-10-producing cells in circulation and in secondary lymphoid tissues, particularly the spleen (80%). Conversely, IL-10-producing B cells were rare. In addition, approximately 10% to 20% of the neutrophils in the blood and spleen expressed MHC II and were IL-10 negative, suggesting that neutrophils could also participate in antigen presentation. *In vitro* investigation of bovine neutrophils revealed that exposure thereof to OO extract increased IL-10 and MHC II expression in these cells in a dose-dependent manner, consistent with IL-10+/MHC II+ neutrophils detected in cattle shortly after experimental OO infection. Co-culture of untreated neutrophils with anti-CD3 antibody (Ab)-stimulated CD4+ T cells led to enhanced T cell activation; also, IL-10 depletion with neutralizing Ab enhanced the stimulatory function of neutrophils. OO extract depressed neutrophil stimulation of CD4+ T cells in the presence of IL-10-neutralizing Ab, suggesting that OO utilizes both IL-10-dependent and independent mechanisms to manipulate the bovine immune response. Finally, contact and viability were required for T cell-stimulatory neutrophil function. This report, to the best of our knowledge, is the first to demonstrate that neutrophil-derived IL-10 is directly involved in T cell regulation in cattle. Our data suggest that neutrophils and neutrophil-derived IL-10 are co-opted by nematode parasites and other pathogens to attenuate host immune responses and facilitate pathogen survival.

## Introduction

Neutrophils are one of the immune system’s first responders and protect the host against invading pathogens through phagocytosis and the production of antimicrobial molecules and reactive oxygen species (ROS)^1–3^. Neutrophils also modulate the adaptive immune response by recruiting other immune cells such as dendritic cells (DCs) to the infection site^4^. Increasing evidence suggests direct neutrophil involvement in regulating T cell functions through the production of IL-10^5^. In mice, neutrophils produce large quantities of IL-10 following mycobacterial stimulation that dampens local lung inflammation and suppresses control of the mycobacteria during the chronic phase of the infection^6^. In addition, the extracellular and intracellular pathogens *Yersinia enterocolitica* and *Salmonella* induce IL-10 production in neutrophils shortly following infection^7^. *Trypanosoma cruzi* stimulated neutrophil production of IL-10, which inhibits T cell proliferation and IFNγ production^8^. In humans, systemic amyloid A-1 induces IL-10-producing neutrophils in melanoma patients, thereby inhibiting tumor-specific CD8+ T cell functions *in vitro*^9^. IL-10 production in human neutrophils can be induced by pathogens such as *Paracoccidioides brasiliensis*^10^ or by lipopolysaccharide (LPS)-stimulated regulatory T cells through direct, cell-to-cell contact^11^. In cattle, IL-10 mRNA expression is upregulated when animals become infected with the abomasal parasite *O. ostertagi* (OO)^12^ or when chronically infected with Staphylococcus aureus, one of the causative agents of mastitis^13^. In addition, increased IL-10 mRNA expression has been detected in maternal neutrophils on the day of calving^14^. Despite these findings, functional IL-10 production by bovine neutrophils and its role in T cell activation remain unknown^15,16^.

Ostertagiasis, caused by the nematode parasite OO, is among the most economically important gastrointestinal (GI) nematode parasites of cattle in temperate regions worldwide. Considerable effort has been invested in developing a vaccine against OO over the last decades^17–20^; however, the inability to identify viable vaccine targets coupled with a limited understanding of the host immune response to nematode infections^21–23^ have contributed substantially to the multitude of vaccine failures. A protective vaccine against *Taenia ovis*, albeit a cestode of sheep, has been developed^24,25^, offering promise for a future vaccine against nematodes like OO.

Parasites have evolved sophisticated strategies for evading the host immune response, many of which are not well understood^26^. In cattle infected with OO, antigen-specific CD4+ T cell and B cell responses are induced^27–30^; however, adaptive immunity is slow to arise and relatively weak compared to immune responses against other GI parasitic nematodes^31^. Studies based on mRNA quantification indicate that the immune response to a primary OO infection in cattle differs from the classical T_h_2 paradigm^30,32,33^, instead favoring a T_h_0 cytokine profile (characteristic of both T_h_1 and T_h_2 responses) over the course of the infection^34^. Specifically, the cattle immune response to OO is characterized by an increase in the T_h_1 cytokine IFNγ alongside increases in T_h_2 cytokines IL-4, IL-5, and IL-10^12^. This mixture of T_h_1 and T_h_2 features suggests the involvement of regulatory players such as regulatory T cells (Tregs), regulatory B cells (Bregs) that might be IL-10+ and CD25+^35,36^, and other immune cells, including neutrophils. The enigma deepens when differences in host responses occur among animals given large bolus infections of OO versus smaller trickle infections (12).

B cells amass in the abomasal draining lymph nodes (DLNs) of cattle chronically infected with OO^37,38^. We first speculated that a population of Bregs were present in the DLNs^37^, but recently discovered that bovine neutrophils respond to whole worm extracts of OO by forming neutrophil extracellular traps (NETs), suggesting that neutrophils respond directly to parasites and/or their products^39^. In this report, we investigated IL-10-producing neutrophils in OO-infected cattle and their potential participation in the activation of CD4+ T cells.

## Results

### Bregs in cattle

**Figure 1:**
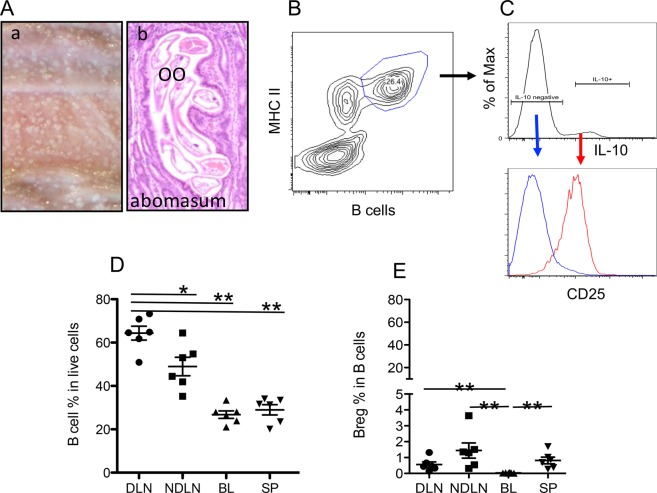
Characterization of regulatory B cells (Bregs) in cattle. *O. ostertagi* (OO)-infected cattle were examined for the presence of pathology and parasites in (**A**), OO-exposed (grass-fed) cattle were used for the analysis of IL-10+/CD25+ Bregs (**B**–**E**). (**A**) Representative gross (**A**-a) and microscopic (**A**-b) pathologies of bovine abomasum experimentally infected with OO. Infected abomasum showed typical nodular pathology (nodules) and OO larvae present in abomasal gastric glands. (**B**,**C**) Gating in flow cytometry on B cells and representative flow cytometry analysis of the expression of IL-10 and CD25 in B cells. (**D**) Analysis of B cell distribution in secondary lymphoid tissues and blood in beef cattle raised on pasture (grass-fed). (**E**) IL-10+/CD25+ B cells or Bregs in total B cells. LN, Lymph nodes; DLN, draining LN; NDLN, non-draining LN; BL, blood; SP, spleen. Data in (**D**,**E**) are expressed as mean ± SEM. *P < 0.05; **P < 0.01; ***P < 0.001; NS: not significant. Data in (**D**,**E**) were analyzed by Mann-Whitney *t*-test.

Ostertagiasis is highly prevalent in cattle raised on pasture under temperate conditions^40,41^. Abomasal tissues harvested from cattle experimentally infected with OO presented with widespread, volcano-like nodules on their surface mucosa (Fig. 1A-a)^37^. Significant immune cell infiltration was evident in tissues surrounding the abomasal gastric glands where the OO L4 resided (Fig. 1A-b). Earlier studies showed increased recruitment of B cells to the DLNs of infected abomasa in grass-fed cattle^37,38^. The present study confirmed a drastic expansion of B cells in DLNs compared to non-DLNs, blood, and spleen (Fig. 1D). Monoclonal antibodies recognizing surface IgM, CD21 (B cell marker for cattle)^42,43^, and MHC II defined the identical B cell population (Supplementary Fig. S1), so surface IgM antibody was used for B cell identification. We speculated that some of these B cells were Bregs influencing immune reactivity by producing IL-10^35,36^. However, intracellular staining with anti-bovine IL-10 antibody (Ab)^44–46^ revealed but a small fraction of IL-10+ B cells (Fig. 1B,C). These IL-10-producing B cells were also high in CD25 expression, suggesting a Breg phenotype (Fig. 1B,C, and data not shown). However, they constituted only 1% of total B cells in the secondary lymphoid tissues and were virtually undetectable in the blood (Fig. 1E).

### IL-10-producing neutrophils in secondary lymphoid tissues and blood in cattle

**Figure 2:**
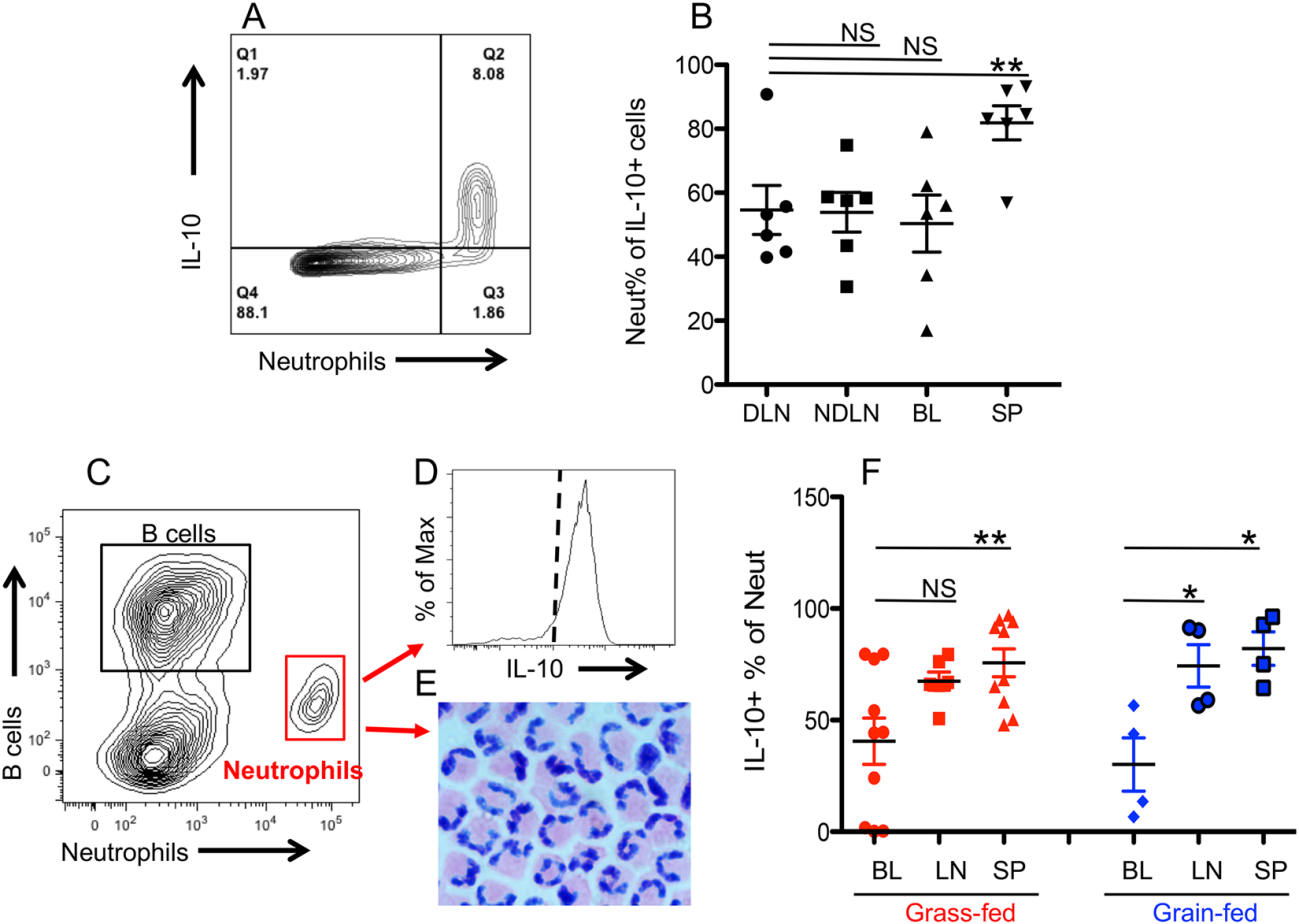
IL-10-producing neutrophils in cattle. Samples were collected from grass-fed (**A**–**E**) and grain-fed cattle (**F**). (**A**,**B**) Analysis of neutrophils in total IL-10-producing cells. (**C**,**D**) Defining neutrophils based on antibody (**C**) and representative histogram on IL-10 expression (**D**). (**E**) Representative morphology of sorted neutrophils stained by Giemsa stain. (**F**) Comparison of IL-10-producing neutrophils between grass-fed and grain-fed cattle. Data in (**B**,**F**) were analyzed by Mann-Whitney *t*-test.

Potential sources of IL-10 in OO-infected animals were evaluated. The existence of nematodes on the Wye Angus farm was indicated by egg-shedding rates in the enclosed calf herd^47^ and in our previous report^37^. Results showed that the majority of the IL-10+ population comprised neutrophils, rather than B cells, macrophages or monocytes, or T cells (Fig. 2A,B, and data not shown). This is consistent with previous findings showing that bovine neutrophils respond to both OO extract and whole parasites^39^. The highest proportion of IL-10-producing neutrophils was found in the spleen (~80%) and was significantly higher (*P* < 0.01) than that found in the LNs or in the blood (*P* < 0.05) (Fig. 2B). Neutrophils sorted with an anti-neutrophil Ab (Clone #CH138A, Fig. 2C) demonstrated a typical segmented nuclear morphology (Fig. 2E), and a large proportion was IL-10-producing regardless of origin (lymphoid tissues or blood) or host diet (grass-fed or grain-fed) (Fig. 2F and data not shown).

### MHC II+ neutrophils in cattle

**Figure 3:**
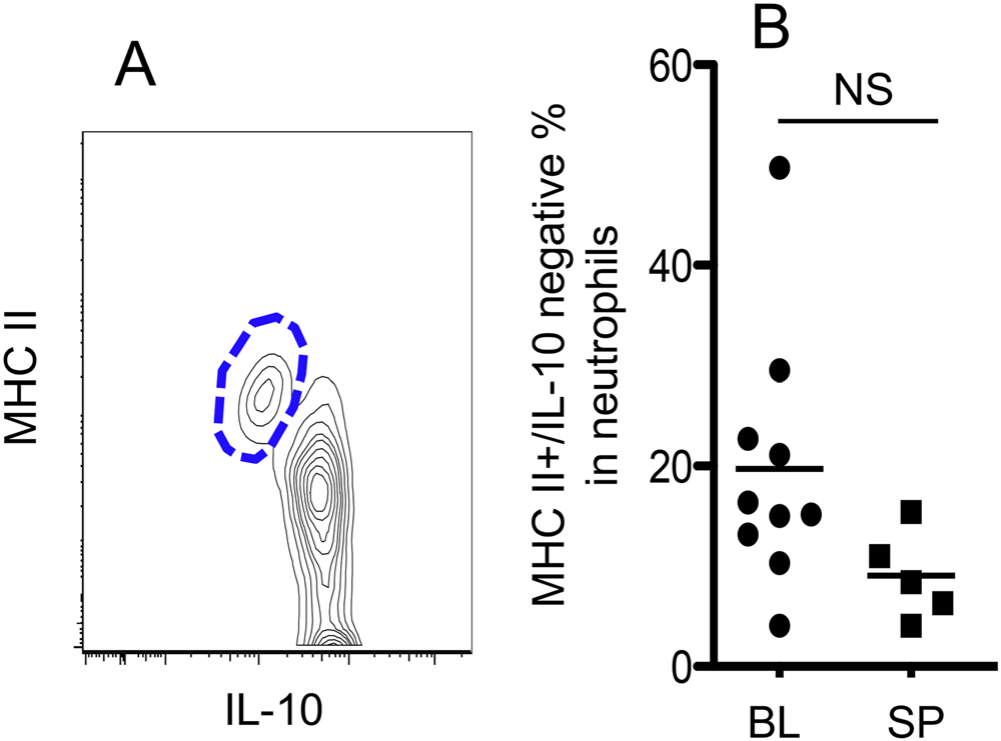
MHC II+/IL-10- neutrophils in cattle. Samples were collected from grass-fed cattle. (**A**) Gating on MHC II+/IL-10- in neutrophils. (**B**) Comparison of MHC II+/IL-10- neutrophils derived from blood and spleen. Data represent results from at least three independent experiments. Data in (**B**) were analyzed by Mann-Whitney *t*-test.

In grass-fed cattle, we observed a small population of neutrophils expressing intermediate levels of MHC II relative to B cells (Fig. 1B). These MHC II+ neutrophils were IL-10 negative and constituted between 10% and 20% of total neutrophils in the spleen and blood (Fig. 3A,B). These data suggest that some neutrophils may function as antigen-presenting cells to stimulate T cell activation.

### Bovine neutrophils are viable for at least 16 hours *in vitro*

**Figure 4:**
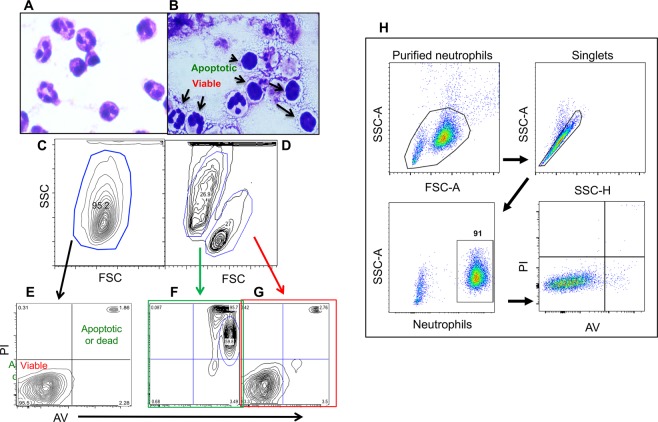
Bovine neutrophil viability in culture. Neutrophils were purified from the blood of 12 to 18-mo grass-fed cattle and cultured *in vitro* for 16 h (**A**,**C**,**E**) or 30 h (**B**,**D**,**F**,**G**). (**A**,**B**) Morphology of cultured neutrophils, showing viable neutrophils cultured for 16 h and partially viable neutrophils cultured for 30 h. Magnification 1000X under light microscopy. (**C**,**D**) Representative flow cytometric profiles of neutrophils using side scatter (SSC) and forward scatter (FSC). (**E**–**G**) flow cytometric detection of apoptosis of neutrophils cultured for 16 h (**E**) and 30 h (**F**,**G**). (**H**) Validation of neutrophil purification. Neutrophils were purified from peripheral blood using centrifugation and red cell lysis was performed as described previously^39^ and examined for general purity using neutrophil antibody and viability assays. PI, propidium iodide; AV, annexin V.

T cell priming (both CD4+ and CD8+) requires direct and stable interactions between T cells and DCs in the LNs for a minimum of two hours and can take up to a full day^48^. We first tested the longevity of bovine neutrophils by culturing the cells for 16 or 30 hours at 37 °C^39^. Those from the 16 hour cultures displayed a healthy scatter plot, multi-lobed nuclear morphology (Fig. 4A,B), and were annexin V (AV) and propidium iodide (PI) negative, indicating viability (Fig. 4E) comparable to neutrophils before culture (Fig. 4H). Approximately 50% of the neutrophils from the 30 hour cultures remained AV/PI negative; the remainder became smaller in size as demonstrated by Forward Scatter (FSC) (Fig. 4D) and displayed AV/PI positive staining consistent with apoptosis (Fig. 4D,F,G)^49^. Morphological analysis further indicated that neutrophils cultured for 30 hours presented as a mixture of viable cells and apoptotic cells with condensed and rounded nuclei^50^ (Fig. 4B) indicating adequate survival time to test their regulatory effects on CD4^+^ T cells *in vitro*.

### *O. ostertagi* extract regulates IL-10 expression in neutrophils

**Figure 5:**
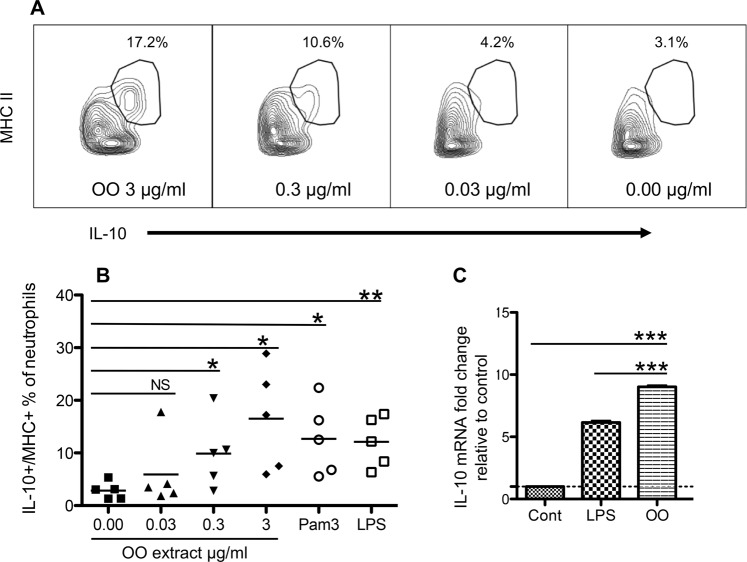
OO extract induces MHC+/IL−10+ neutrophils *in vitro*. Neutrophils were purified from the blood of 12 to 18-mo grass-fed cattle and cultured for 16 h with increasing concentrations of OO extract. Pam3 and LPS were used as positive controls. (**A**) Representative gating of IL-10- and MHC II-expressing neutrophils. (**B**) Comparison of MHC II+/IL-10+ neutrophils induced by medium alone, increasing concentrations of OO extract, Pam3, and LPS. Dose-dependent effects of OO extract on induction of IL-10+MHC II+ neutrophils were analyzed by linear regression (*P* <0.01 as deviation from zero significant). (**C**) Cells were similarly stimulated as in (**B**) and harvested for analysis of IL-10 mRNA expression using quantitative real time PCR. CONT, control; Pam3, Pam3CSK4; LPS, bacterial lipopolysaccharide. Data in (**B**,**C**) were analyzed by paired Student’s *t*-test.

Purified blood neutrophils were cultured in the presence or absence of OO extract at different concentrations. Results showed that in the presence of OO extract, IL-10 production in neutrophils increased in a dose-dependent manner (*P* < 0.01), and that the IL-10-producing neutrophils also expressed MHC II (Fig. 5A,B). This result was corroborated by the upregulation of IL-10 mRNA in neutrophils treated with OO extract (Fig. 5C), suggesting a transcription burst induced by parasite products^51^. Toll-like receptor (TLR) ligands (LPS for TLR4 and Pam3CSK4 for TLR2) caused a similar induction of IL-10+/MHC II+ neutrophils.

### IL-10+/MHC II+ neutrophils detected in cattle shortly after experimental infection with OO

**Figure 6:**
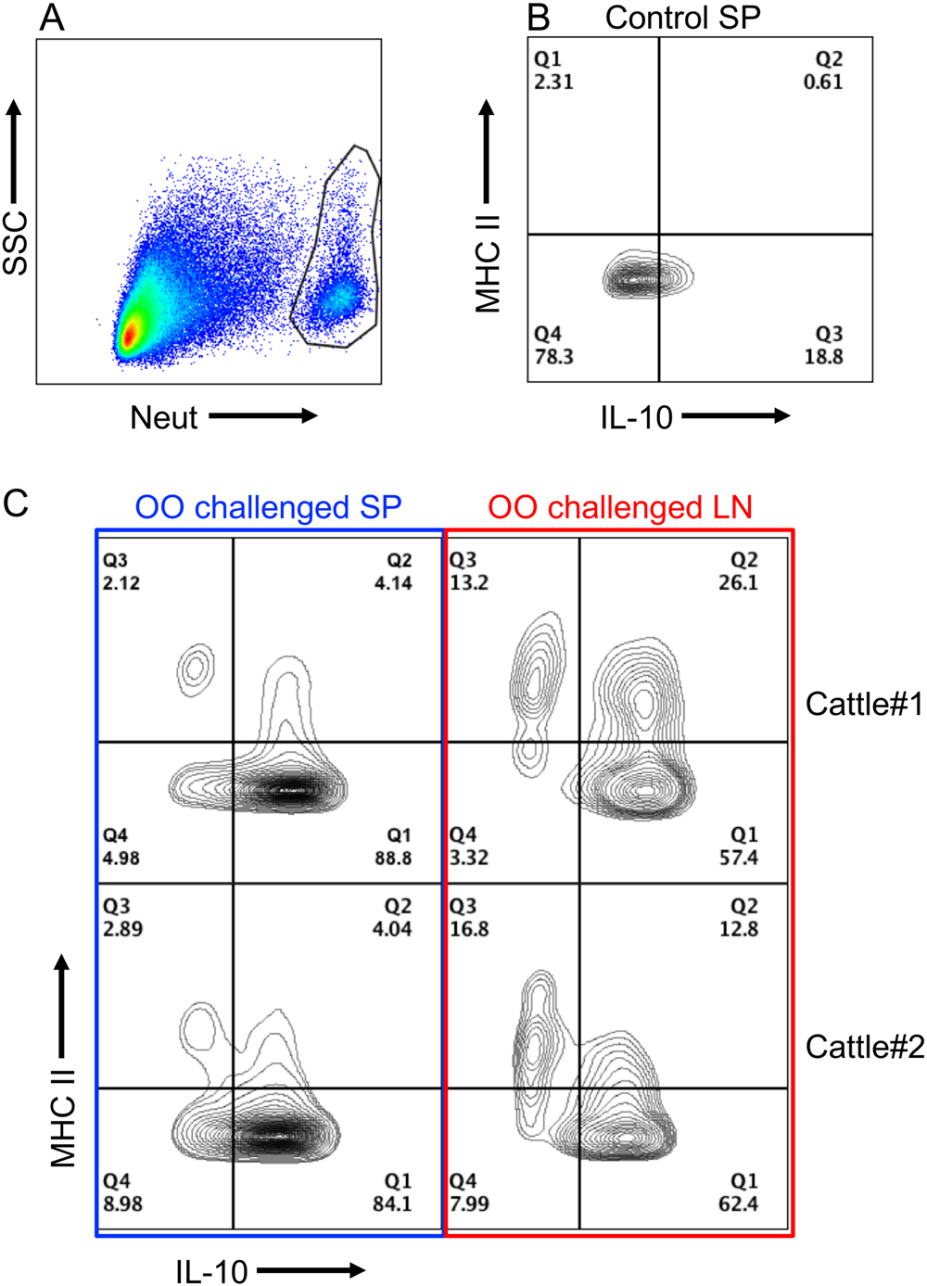
IL-10+/MHC II+ neutrophils detected in cattle shortly after experimental infection with OO. Helminth-free, 5–7 mo Holstein cattle were orally inoculated with 200,000 infective OO L3 and euthanized at 11 days post-infection. Control cattle were age-matched and uninfected. Tissue samples were collected at necropsy. (**A**) Gating on neutrophils using Ab. (**B**) Neutrophil expression of IL-10 and MHC II in control cattle. (**C**) Detection of IL-10-producing/MHC II-expressing neutrophils in cattle experimentally infected with OO.

OO extract induced IL-10+/MHC II+ neutrophils *in vitro* (Fig. 5); conversely, in grass-fed cattle maintained on pasture with repeated natural re-infections, and thus harboring chronic OO-infections, MHC II+ neutrophils failed to produce IL-10 (Fig. 3). It is possible that OO induces IL-10+/MHC II+ neutrophils only upon primary infection or during the early stages of a primary infection. To test this possibility, two weaned, helminth-free calves were experimentally infected with OO for 11 days. IL-10+/MHC II+neutrophils were detected in the spleen and inguinal LNs during this period (Fig. 6C), but not in the spleen of uninfected calves (Fig. 6A,B). OO infection also induced MHC expression in the IL-10 negative population (Fig. 6C, Q3), a pattern that diverged from the *in vitro* results shown in Fig. 5.

### Neutrophils activate CD4+ T cells, and depletion of IL-10 further enhances T cell activation

**Figure 7:**
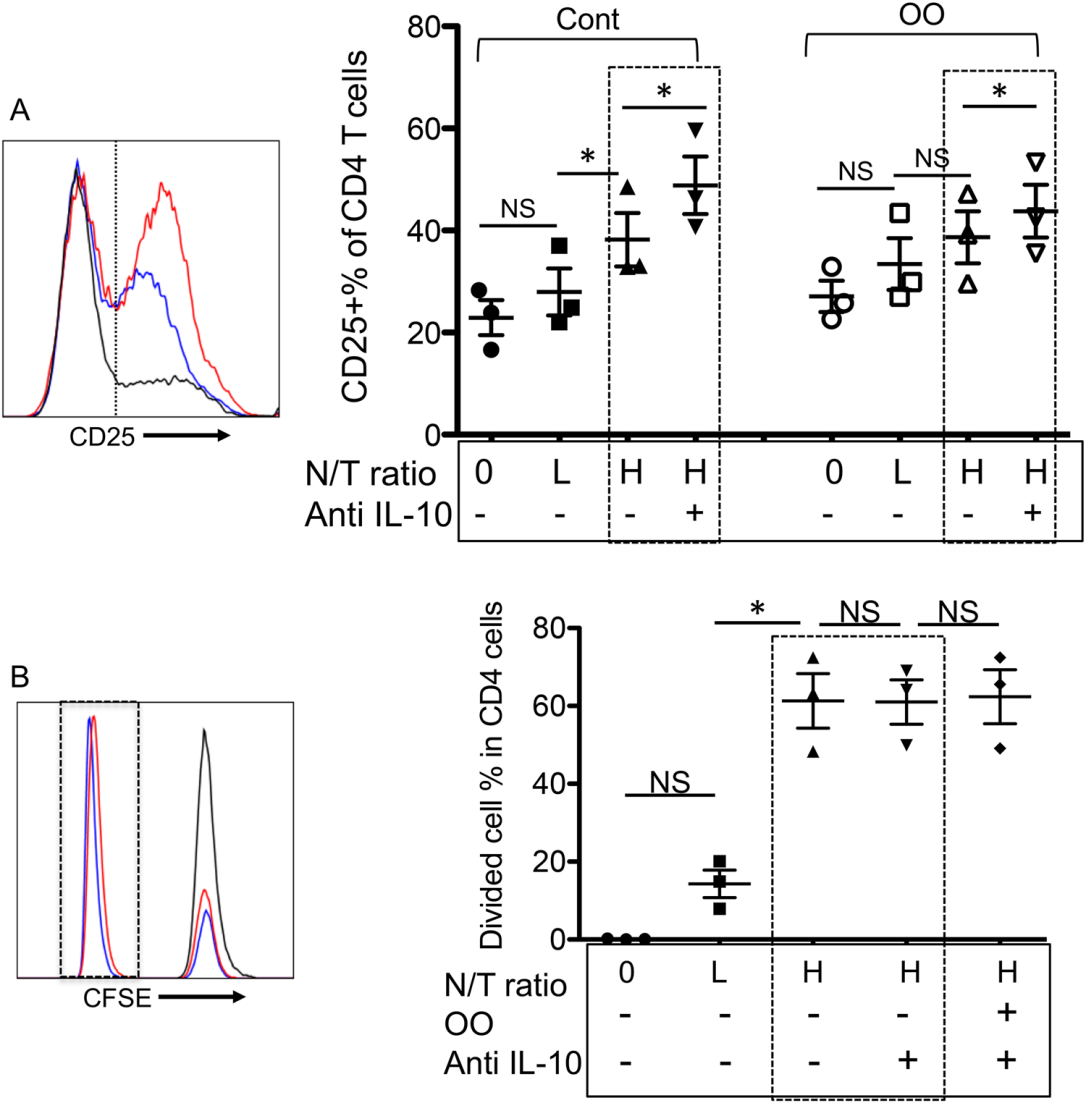
IL-10 suppresses neutrophil-mediated activation of CD4+ T cells. Plates (24-well) precoated with anti-bovine CD3 Ab were used in this experiment. CD4+ T cells (sorted, then CFSE-labeled) and neutrophils from the same animal (n = 3, grass-fed cattle) were co-cultured at different ratios in the presence or absence of OO extract and/or IL-10-neutralizing antibodies. The labels of “L” or “H” below the X-axis indicate the ratio of neutrophils to CD4+ T cells (N/T), with L indicating 1:1, and H indicating 10:1. Cells were cultured for 3.5 days prior to staining for flow cytometry analysis. (**A**,**B**) Gating and analysis of neutrophil effects on CD25 expression (**A**) and proliferation (**B**) on CD4+ T cells. Black-dotted squares indicate the effects of IL-10 blockage. Data represent results of two independent experiments. Data in (**A**,**B**) were analyzed by paired Student’s *t*-test.

To test if neutrophils could influence T cell activation, sorted bovine CD4+ T cells (Supplementary Fig. S2) (confirmed CD25 negative) were stimulated with plate-coated, anti-bovine CD3 Ab and co-cultured with purified neutrophils from the same cattle at varying ratios. After 3.5 days in culture, cells were stained and analyzed with flow cytometry by gating on CD4+ T cells using an Ab to exclude neutrophils (Supplementary Fig. S3). CD25 expression on CD4+ cells was enhanced by neutrophils at a neutrophil-to-T cell ratio (N/T) of 10 to 1, illustrating significant neutrophil-mediated T cell activation (*P* < 0.05) (Fig. 7A). In the presence of OO extract, the percent CD25 differences between low (1:1) and high (10:1) ratios were not significant. To compare neutrophil stimulating capacity to that of conventional dendritic cells (cDC), MHC II+/CD11c+/Lin- cDCs were purified (Supplementary Fig. S5), using a well-established protocol by the Golde group^52^. Results showed that cDCs significantly enhanced CD4+ T cell CD25 expression and proliferation at a T cell-to-cDC ratio of 10:1 (Supplementary Fig. S5). However, the effects of neutrophils at the same frequency or 10 times higher (as in neutrophil “L” in Fig. 7) were not significant (Supplementary Fig. S5). Neutralization of IL-10 using neutralizing IL-10 Ab (Clone #CC320) led to significant upregulation of CD25 on CD4+ T cells co-cultured with neutrophils with or without OO extract (Fig. 7A, black dotted squares), where OO extract appeared to reduce the stimulatory capacity of neutrophils when IL-10 was neutralized (Fig. 7A). It has been reported that anti-CD3 stimulation of CD4+ T cells can induce Tregs in both mice and humans^53–55^. However, only a small fraction of Tregs was detected in anti-CD3 stimulated bovine CD4+ T cells (Supplementary Fig. S4). The population of Treg cells induced by anti-CD3 is small or undetectable, but this might only reflect the *in vitro* effect of anti-CD3 stimulation, and not Treg induction in pathogen-infected cattle^56,57^. Regulatory factors, including pathogen-derived antigens and/or pathogen-associated molecular pattern molecules (PAMPs) and host regulatory cytokines such as TGF-β should be collectively responsible for the induction of Tregs^58^. Interestingly, bovine γ/δ T cells have been reported to include a major Treg population, indicating the complexity of origin and regulation of Tregs^59^. These data suggest that OO extract downregulates neutrophil functions through secondary mechanisms in addition to IL-10 production. Adding OO extract to CD3-stimulated CD4+ T cells did not significantly alter CD25 expression (Fig. 7A), nor did it affect the rate of CD4+ T cell division in the presence or absence of IL-10 (Fig. 7B). These data suggest that bovine neutrophils regulate CD4+ T cells in a multifaceted manner and that IL-10 and pathogens may synergistically manipulate the stimulatory effect of neutrophils on CD4+ T cells. Interestingly, we found that anti-CD3 stimulated CD4+ T cells significantly reduced IL-10 expression in co-cultured neutrophils after a 16-hour co-culture (Fig. S6). In the future, we plan to investigate this finding further to assess whether intracellular IL-10 reduction in neutrophils is due to IL-10 secretion or downregulation on transcription or translation.

### Effects of neutrophils on CD4+ T cells are contact- and viability-dependent

**Figure 8:**
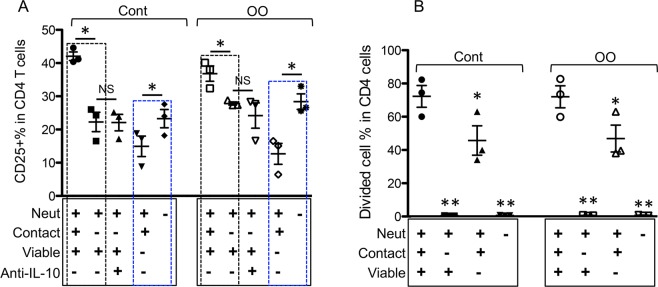
Effects of neutrophils on CD4+ T cells are contact- and viability-dependent. CD4+ T cells (sorted, then CFSE-labeled) and neutrophils (purified) from the same animal (n = 3, grass-fed cattle) were co-cultured in anti-bovine CD3 Ab-coated plates at a high N:T ratio (10:1). CD4+ T cells were either in direct contact with neutrophils in a conventional 24-well plate or separated from neutrophils using a transwell plate. Dead neutrophils were obtained by fixing purified viable neutrophils with paraformaldehyde for 15 min at 4 °C and recounted for plating at the same ratio as viable neutrophils. Cells were cultured for 3.5 days and analyzed for CD25 expression (**A**) and cell proliferation/division (**B**) in CD4+ T cells. Data were analyzed by paired Student’s *t*-test. Black-dotted inset in (**A**) indicates the effect of cell-to-cell contact, and the blue-dotted insets in (**A**) illustrate the inhibitory effects of fixed neutrophil on CD25 expression in CD4+ T cells.

To test if contact between neutrophils and T cells is required for T cell activation, transwell plates were used to prevent direct neutrophil contact with CD4+ T cells. The removal of neutrophil-T cell contact resulted in the complete elimination of neutrophil effects on CD4+ T cell proliferation and a significant reduction in CD25 expression in the presence and absence of OO extract (Fig. 8A,B). Of note, when neutrophils were not in direct contact with CD4+ T cells, blockage of IL-10 did not affect CD4+ T cell activation (Fig. 8A). We further examined if viability was required for neutrophil function by co-culturing anti-CD3-stimulated CD4+ T cells with live or dead (paraformaldehyde-fixed) neutrophils allowing for direct cell contact. Killed neutrophils induced significantly less activation of CD4+ T cells than their live counterparts, as demonstrated by reduced CD25 expression (Fig. 8A dotted blue squares). However, the proportion of dividing cells was significantly enhanced by dead neutrophils (Fig. 8B). Our results suggest that neutrophil regulation of CD4+ T cell activation requires cell-cell contact and is viability-dependent.

## Discussion

In this report, we demonstrate that IL-10 production is an important feature of bovine neutrophils regardless of farming style (grass-fed vs. grain-fed) or parasite (OO) exposure. OO extract upregulated the production of IL-10 and expression of MHC II by neutrophils *in vitro*, and these cells were detected in cattle as soon as 11 days after OO infection. Neutrophils also directly enhanced the activation of CD4+ T cells in a contact- and viability-dependent manner, whereas the neutralization of IL-10 increased neutrophil stimulation of CD4+ T cells.

Bovine neutrophil function is influenced by physical conditions and pathogens^60^. Neutrophils with abnormal functions have been associated with the development of diseases such as puerperal metritis and subclinical endometritis^61^. Neutrophils from the early postpartum period demonstrate reduced chemotaxis, phagocytosis, myeloperoxidase activity^62–64^, and decreased ROS production^62,63,65^. This functional shift by neutrophils might be related to changes in their protein compositions; approximately 40 neutrophil-specific proteins are differentially expressed in periparturient cows^66^. Bovine neutrophil function can be enhanced by the injection of recombinant granulocyte-colony-stimulating factor into cows seven days before and 24 hours after calving; this treatment regimen increases neutrophil counts in the blood and enhances myeloperoxidase release^67,68^. Enhanced neutrophil function is also associated with a reduced incidence of clinical mastitis^69^. Results from this study are consistent with the notion that bovine neutrophils are unique in their functional plasticity and can be regulated by pathogens like OO and their secreted products.

The elevation of IL-10 mRNA expression has been observed in neutrophils on the day of calving^14^ and in milk neutrophils of cattle infected with *S. aureus*^13^. Our data confirm these findings and provide new evidence to support the production and regulation of neutrophil-derived IL-10. Of note, the IL-10-producing/CD25+ B cell population is but a small fraction of total B cells, calling into question its regulatory capacity.

The mechanistic underpinnings of neutrophil-T cell interactions in cattle remain unknown. We speculate that IL-10 production is intrinsic to bovine neutrophils and can be regulated by pathogens like OO. The small population of MHC II-expressing neutrophils found in the spleen and blood also suggests that some neutrophils act as antigen-presenting cells in cattle. Although the stimulatory function of neutrophils is weaker than that of cDCs (Supplementary Fig. S5), the large numbers of neutrophils in circulation and at the site of infection suggest an important role in immunity. We surmise that a small percentage of constitutive, MHC II+/IL-10- neutrophils may participate in disease resistance by aiding T cell activation, whereas MHC II+/IL-10+ neutrophils are induced early in infection and are limited in their capacity to activate T cells. Consistent with these findings, a recent report demonstrated the presence of MHC II+ neutrophils in the lymph nodes of healthy humans and mice^70^. Nematode infections induce rapid recruitment of neutrophils to the site of infection^71^. In livestock, *Haemonchus contortus* infection triggers neutrophil infiltration into the abomasum, possibly by nematode-derived chemotactic factors^72–75^. However, we found that while chronic OO infection in the abomasal mucosa was accompanied by a massive intrusion of mast cells and B cells, it did not elicit neutrophil infiltration^37^. Lymph nodes are uniquely equipped to facilitate activation of adaptive immune responses by bringing DCs and macrophages in proximity to naive T cells, enabling effective priming and antigen presentation^76^. DLNs are thereby ideal tissues wherein pathogens can regulate immune responses. We speculate that IL-10-producing MHC II+ neutrophils are induced in abomasal DLNs in the days following an OO priming infection, then quickly decline to undetectable levels in cattle with extended and repeated OO exposure. We are currently testing this hypothesis by investigating neutrophil and B cell responses in cattle in the early stages of OO infection.

Activation of naive CD4+ T cells requires three signals: antigen (S1), co-stimulation (S2), and instructive cytokines (S3)^4^. The stimulatory function of neutrophils might have provided S2 and/or S3 to anti-CD3 stimulated CD4+ T cells in our *in vitro* system. Our discovery that dead, intact neutrophils inhibited CD25 expression suggests that the surface molecules on bovine neutrophils are inhibitory, and fast neutrophil turnover may be the reason that dead neutrophils persist in their inhibitory capacity and keep ongoing inflammation at bay^2^. Considering the opposing effects of live versus dead neutrophils, we postulate that neutrophil viability is critical for stimulating (providing S2 and/or S3) T cells upon contact, possibly via the release of regulatory molecules. Alternatively, neutrophil viability may be necessary for the constitutive expression of stimulatory molecules on the cell surface.

Based on the *in vitro* data in this report, we hypothesize that bovine neutrophils possess complex regulatory machinery for T cell manipulation. This contact-dependent activity necessitates that neutrophils be in close physical proximity to T cells, which in turn would prevent the misfiring of T cells at secondary locations and avert autoimmune pathologies. Contact-dependent effects of neutrophils are further manipulated by the presence of pathogens such as OO, which could induce IL-10 production in neutrophils and render the neutrophil-T cell interaction less effective. The presence of IL-10 could also lead to suboptimal antibody or T cell responses, thereby facilitating OO survival in the abomasal mucosa. As demonstrated in mice, the initial primary immune response is often the determining factor for the success of the host immune response and infection resolution. In this case, poor stimulation of T cells at the onset of infection would lead to a suboptimal immune response, thus enabling long-term parasite residency. This impaired immunity could be one reason for the persistence of OO infection in pasture-raised cattle. We believe that IL-10+ neutrophils are able to secrete IL-10, as supported by the effects of IL-10 depletion following exposure to neutralizing Ab. However, this IL-10 secretion was found to be cell-cell contact dependent, as the separation of neutrophils from CD4+ T cells completely abolished any effects from neutrophils, including IL-10 production (Fig. 8A), consistent with the mechanisms of Bregs in human and mice^77,78^.

The presence of IL-10-producing neutrophils in secondary lymphoid tissues in both grass-fed and grain-fed cattle, especially in the spleen, suggests their participation in immune homeostasis as well as infection responses. Neutrophils are present in the lymph nodes and spleens of healthy humans and animals^70^, and function as helper cells to B cells^79^ and natural killer (NK) cells^3^. Future work will test the function of these spleen-resident neutrophils on bovine B cells^79^ and other immune cells.

In summary, our data suggest that bovine neutrophils are critical for CD4+ T cell activation in cattle. Results also indicate that neutrophil-mediated T-cell activation is influenced by IL-10, the production of which is further regulated by pathogens. The effects of bovine neutrophils on CD4+ T cells are both contact- and viability-dependent, which contrasts with paradigms established in humans and mice. A more thorough understanding of the onset of immune responses involving neutrophils is needed to facilitate the development of immune-based control measures in the face of emerging drug resistance in livestock GI nematodes.

## Methods

### Cattle

The Wye Angus herd is a closed herd maintained by the Wye Research and Education Center at the University of Maryland Experimental Station (Queenstown, MD)^80^. All calves were weaned at six months with pasture access before weaning; all animals were exposed to various GI nematode parasites on pasture, including *O. ostertagia*^47^. The calves were dewormed following weaning and then randomly assigned to either the grain-fed or grass-fed group. The grain-fed group was kept on a feedlot with access to a mixed diet consisting of corn silage and shelled corn and soybean supplemented with trace elements^37^. The grass-fed group continued to forage on pastures consisting primarily of alfalfa during the grazing season and was fed bailage during the winter months. Grain-fed cattle reached market weight at 14 months of age, while pasture-raised cattle reached market weight at 20 months of age^80,81^. Helminth-free Holstein cattle were raised and maintained indoors on concrete slabs from birth at the Beltsville Agricultural Research Center (BARC) in Beltsville, MD. Animal Care and Use Protocols were approved by both the BARC (#16–019) and UMD (R-FEB-18-06) Institutional Animal Care and Use Committees. All methods were performed in accordance with relevant guidelines and regulations.

### Experimental infection of cattle, parasite propagation, and OO extract preparation

Two helminth-free Holstein calves 5–7 months of age were orally infected with a single dose of 200,000 infective OO L3 on day 0 and euthanized on day 11 post-infection^82^. At necropsy, blood, spleen, and lymph nodes were collected, and the cells were isolated and used immediately or stored in liquid nitrogen for later use.

OO adult worms were propagated in helminth-free cattle, as described previously^83^. Briefly, 5- to 7-month-old, helminth-free Holstein calves were inoculated with a bolus dose of OO L3 (200,000) on day 0 and euthanized on day 21 for adult worm harvest. At necropsy, the abomasum and its contents were collected for parasite isolation using a modified gel migration method^84^, where abomasal contents containing 1% agar were poured onto Whatman filter papers before solidification and then permitted to hang in chambers filled with PBS to facilitate worm migration from the abomasal debris. Adult parasites were washed twice with cold PBS and immediately homogenized in cold PBS on ice at maximum speed for five pulses 15 sec in length on a Polytron homogenizer (Brinkmann Instrument, Westbury, NY). The homogenate was centrifuged at 20,000 *g* for 30 min at 4 °C, and the soluble extract (OO extract) was stored at −20 °C prior to experimental use.

### Bovine neutrophil isolation

Jugular vein blood was collected from cattle using vacutainers containing EDTA or no additive (Becton Dickinson Vacutainer Systems, Franklin Lakes, NJ). Neutrophils were isolated as previously described^39,85^ with minor modifications. Briefly, blood was transferred to 15 mL conical tubes (Fisher Scientific, Pittsburgh, PA, USA) and centrifuged for 20 min at 1,000 *g* at 4 °C. Following centrifugation, the plasma, buffy coat, and one-third of the red blood cell pellet were discarded. The remaining cells were suspended in 5 mL ammonium-chloride-potassium (ACK) lysis buffer to remove red blood cells. The cell suspension was gently mixed and incubated for 5 min at room temperature (RT). The solution was then centrifuged 10 min at 200 *g* at 4 °C, and the supernatant was decanted. The pellet was washed with 15 mL of calcium- and magnesium-free PBS (CMF-PBS) and centrifuged for 5 min at 850 *g* at 4 °C. ACK treatment was repeated for complete red blood cell lysis. Cells were then washed twice with 15 mL of CMF-PBS and centrifuged for 5 min at 850 *g* at 4 °C. After the final wash, the pellet was suspended in 1 mL of RPMI-1640 lacking phenol red (Gibco, Fisher Scientific, Waltham, MA), and neutrophil concentrations were measured using the trypan blue exclusion method on a hemocytometer. Killed but intact neutrophils were obtained by treating purified neutrophils with 2% paraformaldehyde (Fisher Scientific, Pittsburg, PA) at 4 °C for 15 min, followed by two washes with RPMI-1640 supplemented with 5% FBS. The morphology of the killed neutrophils was confirmed by microscopy, and cells were re-counted, suspended in RPMI-1640 supplemented with 5% FBS, and stored at the same concentration as live neutrophils for use in corresponding experiments. For *in vitro* stimulation of isolated neutrophils, 2 × 10^6^/mL cells were cultured in RPMI-1640 supplemented with 5% FBS (TCB, Tulare, CA). Lipopolysaccharide (LPS; TLR4 ligand) and Pam3Cys (TLR2 ligand) were purchased from Invivogen (San Diego, CA), at concentrations of 5 µg/mL and 100 ng/mL, respectively^86^. The different treatments (OO extract, LPS, and Pam3Cys) were added at the beginning of the stimulation.

### qPCR

Cultured neutrophils were collected and washed twice with 1X PBS, and the resulting neutrophil pellets were lysed with RLT lysis buffer (Qiagen, Germantown, MD). RNA was isolated using the RNeasy Micro kit (Qiagen), which includes DNase treatment. RNA quality was checked on a NanoDrop 1000 spectrophotometer. All RNA concentrations were approximately 40 ng/µL, and 260/280 ratios ranged from 1.96 to 2.08. An aliquot of 400 ng of total RNA was used for first-strand cDNA synthesis using the Thermo Maxima First Strand cDNA synthesis kit (Fisher, Waltham, MA) following the manufacturer’s instructions. Reverse transcription (RT) was performed in an Eppendorf Master Gradient Cycler (Eppendorf, Hamburg, Germany) with three cycles as follows: initialization by one cycle at 25 °C for 10 min, followed by one cycle at 50 °C for 15 min, and inactivation by one last cycle at 85 °C for 5 min and maintained at 4 °C. Synthesized cDNA in 20 µL was diluted to 80 µL with RNase free water to 5 ng of total RNA per microliter diluted cDNA, and 2 µL of diluted cDNA was later used for Quantitative PCR (qPCR) amplification.

qPCR was performed in a C1000 Touch Thermal Cycler (BioRad, Hercules, CA) using the IQ SYBR Green Supermix (BioRad, Hercules, CA) in a 20 µL reaction. Two µL of diluted cDNA template and two primers (Table 1) (final concertation 400 nM) were applied in each reaction. cDNA was amplified using two-step protocols, with initial denaturation at 95 °C for 3 min, 40 repeated steps consisting of 10 sec at 95 °C, and extension at 55 °C for 30 sec. Melting curves were obtained after 40 cycles. All amplification products were analyzed to confirm correct size by gel electrophoresis and then sequenced.

**Table 1:** Primers for bovine IL-10 and GAPDH.

Primer name	Sequence (5′ → 3′)
IL10 Forward	5′-TGT TGA CCC AGT CTC TGC TG-3′
IL10 Reverse	5′-TTC ACG TGC TCC TTG ATG TC-3′
GAPDH Forward	5′-CTC CCA ACG TGT CTG TTG TG-3′
GAPDH Reverse	5′-CCC AGC ATC GAA GGT AGA AG-3′

### CD4+ T cell isolation

Scalpel blades were used to dissect the superficial inguinal lymph nodes located in the mass of fat about the neck of the scrotum and behind the spermatic cord^87^. The lymph nodes were then cut into 2–3 mm^3^ pieces and digested in 5 mL RP10 medium containing 400 U/mL V Collagenase, 0.1 mg/mL DNase, and 2.5 U/mL hyaluronidase at 37 °C for 2 hours^37,88^. The resulting single cell suspension was incubated with FITC-conjugated anti-bovine CD4 (Clone #CC8, BioRad, Hercules, CA), and PE-conjugated anti-bovine CD8 (Clone #CC63, BioRad, Hercules, CA) for 30 minutes at 4 °C, followed by washing twice with medium. The final suspension contained 2.0 × 10^7^ cells per mL. Sorting was gated on a CD4+/CD8- population in a FACSAria II sorter (BD, San Jose, CA), and the purity of sorted CD4+T cells was confirmed to be >92%; these were further stained with anti-bovine CD25 (Clone #LCTB2A, Washington State University (WSU), Seattle, WA) to confirm CD25 negative status using a FACSCanto I (BD, San Jose, CA).

### Conventional dendritic cell (cDC) purification

Approximately 2–4 × 10^8^ PBMCs were isolated from each animal, as described previously^89,90^. Bovine cDCs were sorted from these PBMCs as previously described^52^, which included two steps: depletion of T cells, monocytes, B cells, and NK cells through negative selection using magnetic beads, followed by sorting of cDCs based on staining of the negatively selected fraction. Briefly, PBMCs were suspended in staining buffer and then incubated for 30 minutes on ice with unlabeled mouse monoclonal Abs against CD3 (MM1A), sIgM (BIG73A), CD11b (MM12A), and CD14 (MM61A), all mouse IgG1 from WSU. The cells were then washed and stained with anti-mouse IgG1 conjugated with FITC (Biolegend, San Diego, CA), followed by incubation with anti-FITC magnetic MicroBeads (Miltenyi Biotech, Auburn CA) and passed through LS separation columns attached to a MACS magnet^91^. Cells that did not bind to the column were collected. This fraction was incubated with mouse monoclonal Abs against bovine MHC II (TH16A, IgG2a) and CD11C (BAQ153A, IgM), followed by anti-mouse IgG2a-APC and anti-mouse IgM-BV421 (Biolegend, San Diego, CA). Final cell suspensions were then stained with anti-bovine CD4 conjugated with PE (BioRad, Hercules, CA). The cDCs were sorted based on CD11c+/MHC II+/CD4−.

### Carboxyfluorescein diacetate succinimidyl ester (CFSE) labeling

CFSE labeling was conducted as previously reported^76,92^. Briefly, sorted CD4+ T cells were adjusted to 10 × 10^6^/mL in Hank’s Balanced Salt Solution (HBSS) (Corning, Manassas, VA) and kept in a 37 °C water bath for 15 min. Stock CFSE (9 mM) was diluted to 44 nM using HBSS, and equal volumes of diluted CFSE were mixed with the pre-warmed cells. The mixture of cells and CFSE was incubated in a 37 °C water bath for 5 min and vortexed 3 times during the incubation period. Cells were then washed 3 times with RPM-1640 containing 10% FBS.

### Co-culture of neutrophils and CD4+ T cells

Anti-bovine CD3 (Clone #MM1A, WSU, Pullman, WA) was added to 24-well plates at 10 µg/mL in 250 µL 1X PBS (Hyclone, Logan, UT), as in previous reports^93,94^. Sorted, CFSE-labeled bovine CD4+ T cells were seeded at 2 × 10^5^ per well, and autologous neutrophils (from the same cattle) were added to the wells of T cells at either 1:1 or 10:1 (2 × 10^6^ neutrophils) ratios. In experiments using transwell plates^95^, CD4+ T cells were seeded in the lower chamber to interact with plate-coated anti-bovine CD3 Ab, and neutrophils were placed in a transwell insert with a pore size of 0.4 µm (Greiner Bio-one, Monroe, NC) in 100 µL medium. Neutralizing Ab against bovine IL-10 (CC320, BioRad, Hercules, CA) was added to the medium at 10 µg/mL^59,94^, and the same amount of purified mouse IgG1 (Biolegend, San Diego, CA) was supplemented in the controls. Plates were incubated at 37 °C in an atmosphere of 5% CO_2_ for 3.5 days and then analyzed for CFSE dilution and CD25 expression in CD4+ T cells using flow cytometry.

### Flow cytometry

Antibodies specific to bovine neutrophils (Clone #CH138A), B cells (Clone #BIG73A, recognizing surface IgM), CD21 (Clone #BAQ15A), CD25 (LCTB2A and CACT108A), MHC II (TH16A), CD4 (GC50A), CD8 (CACT130A), and CD69 (KTSN7A) were obtained from the WSU Monoclonal Antibody Center (Pullman, WA). IL-10 Abs (CC318, CC320) were procured from BioRad (Hercules, CA), and all anti-mouse isotype secondary Abs were purchased from Biolegend (San Diego, CA). For IL-10 intracellular staining, surface staining was performed first, followed by permeabilization and intracellular staining with IL-10, as described previously^96^. Annexin V Ab conjugate and propidium iodide (PI) were from an Annexin V staining kit (Biolegend, San Diego, CA)^97,98^. Manufacturer-recommended concentrations for each Ab were used, typically 1.25–10 µg/mL in 100 µL reaction media.

### Statistical analysis

Statistical analyses were performed with Prism 5 (GraphPad Software, Inc., La Jolla, CA); specific details thereof are provided in the figure legends. All data were analyzed by Mann-Whitney or paired Student’s *t*-test. Dose-dependent effects of OO extract on IL-10+/MHC II+ neutrophil induction were analyzed by linear regression (Fig. 5B). Asterisks indicate statistical significance. *P < 0.05; **P < 0.01; ***P < 0.001.

## Supplementary information

Supplementary Information.
