# Supplementary material for: Characterization of IL-10-producing neutrophils in cattle infected with Ostertagia ostertagi
Source: Sci Rep. 2019 Dec 30;9:20292. doi: 10.1038/s41598-019-56824-x (PMC6937330; doi:10.1038/s41598-019-56824-x)
Supplement: Supplementary file 1 — Supplementary Information. [file 41598_2019_56824_MOESM1_ESM.pdf]

Characterization of IL-10-producing neutrophils in cattle infected with *Ostertagia ostertagi*

Lei Li[a] , Hongbin Si[a], Shu-Wei Wu[a], Jonatan Orangel Mendez[a], Dante Zarlenga[b], Wenbin Tuo[b,*], and Zhengguo Xiao[a,*]

[a] Department of Avian and Animal Sciences, University of Maryland, College Park, MD 20742;

[b] Animal Parasitic Diseases Laboratory, USDA/ARS, Beltsville, MD 20705;

*Corresponding authors:

Email addresses: xiao0028@umd.edu (Z. Xiao); wenbin.tuo@ars.usda.gov (W. Tuo)

Supplementary information

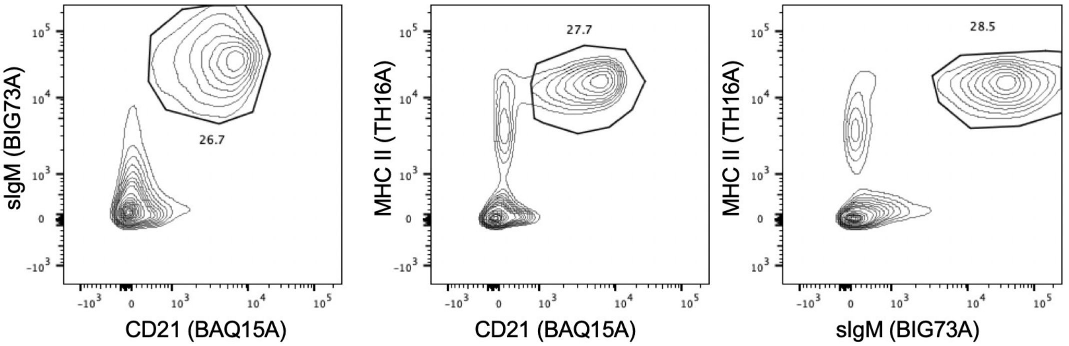

Supplementary Figure S1. Validation of antibodies specific to bovine B cells. Bovine PBMCs were purified and then stained with three mouse monoclonal antibodies against bovine B cell markers: clones BIG73A, TH16A, and BAQ15A recognizing surface IgM, MHC II, and CD21, respectively. Contour plots show populations stained positive for 2 out of 3 antibodies in all three combinations.

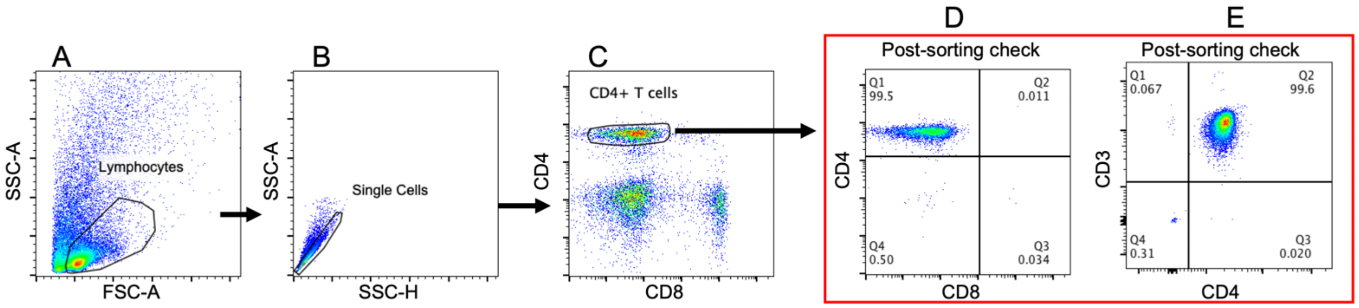

Supplementary Figure S2. Gating strategy for CD4+ T cell sorting. (A) Single cell suspensions of inguinal lymph nodes were stained with anti-bovine CD4 and CD8 antibodies (direct conjugates). CD4 T cell sorting was based on CD4+/CD8- (C) from singlets in lymphocyte population (B). (D) Representative post-sorting purity check. (E) Sorted CD4 T cells were further stained with anti-bovine CD3, and were confirmed to be CD4+/CD3+.

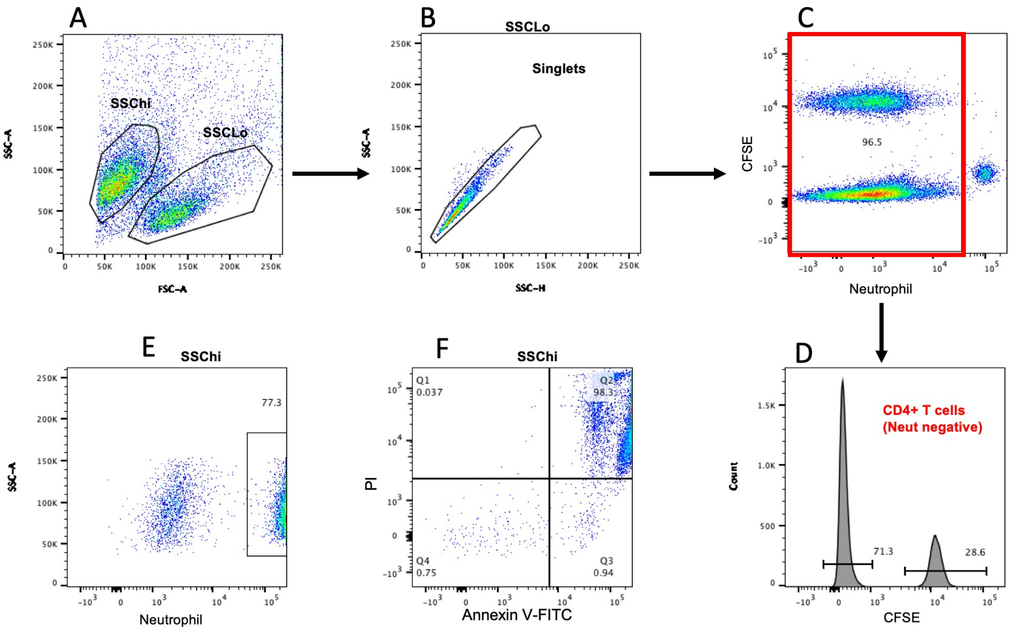

Supplementary Figure S3. Gating strategy for CD4+ T cells in neutrophil/CD4+ T cell co-culture. Cells were harvested and stained after 3.5 days in culture. First, SSC low (SSCLo) were gated (A), and single cells (B) were further gated on neutrophil marker negative (red square gate in C). The CD4+ T cell population is shown in (D) for comparison of CD25 expression and CFSE dilution. Most of the SSC high cells in (A) were apoptotic neutrophils, as indicated in (E) and (F).

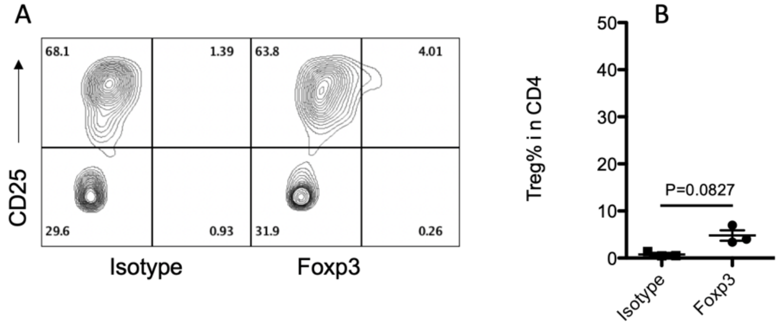

Supplementary Figure S4. Anti-CD3 antibody alone does not significantly induce regulatory T cells (Tregs). Sorted bovine CD4+ T cells were stimulated with anti-bovine CD3 antibody for 3.5 days, and were stained for the expression of CD25 (surface) and Foxp3 (intracellular). Data in (A) represent flow cytometric plots from the same sample stained with anti-CD25 antibody and anti-Foxp3 antibody or isotype control for anti-Foxp3. Data in (B) were analyzed by paired Student's *t*-test.

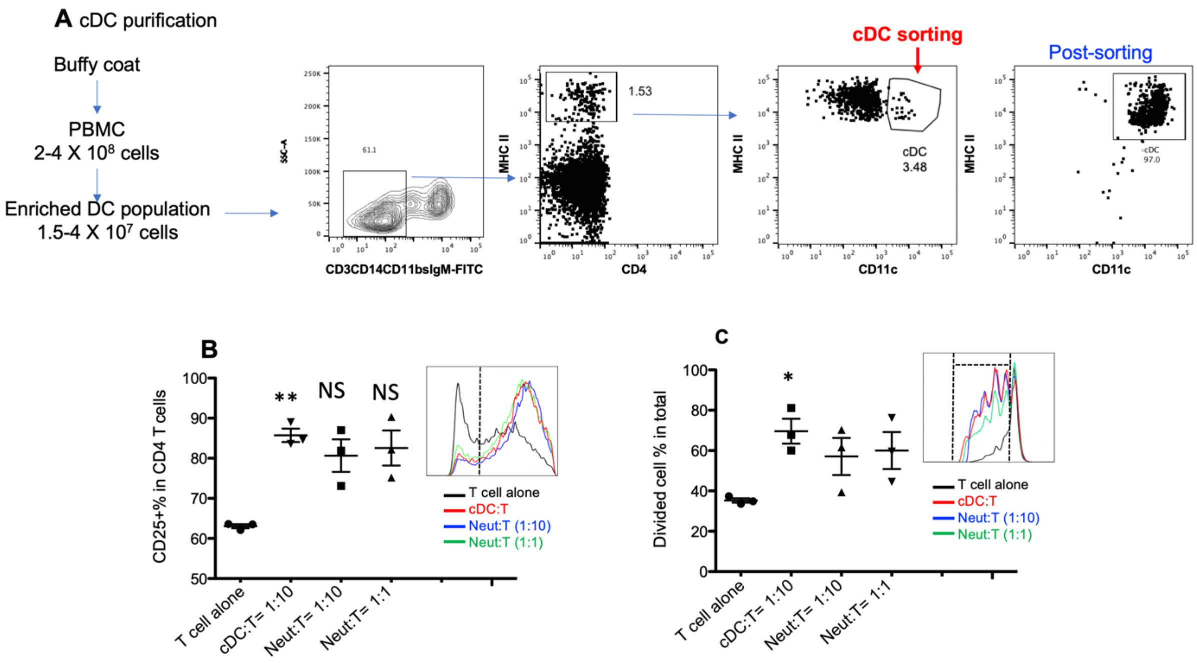

Supplementary Figure S5. cDCs enhance the activation of anti-CD3-stimulated CD4+ T cells. cDCs were purified from bovine peripheral blood (three cattle), as indicated in (A). CD4+ T cells sorted from the same cattle were labeled with CFSE and stimulated by plate-coated anti-bovine CD3 antibody. Purified cDCs or neutrophils (from the same cattle) were added to autologous CD4+ T cells at the beginning of the culture, followed by incubation for 3.5 days. After co-culture, CD4+ T cells were analyzed for CD25 expression (B) and CFSE dilution to indicate T cell proliferation (C). The inserts in (B) and (C) represent histograms demonstrating gating.

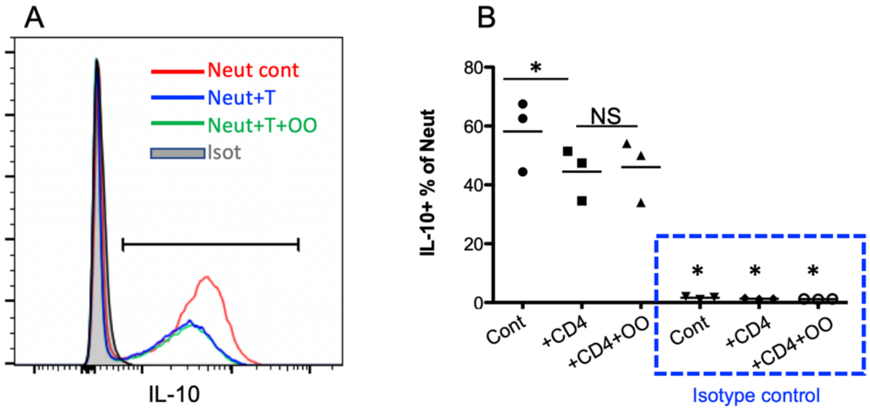

Supplementary Figure S6. Activated CD4+ T cells affect IL-10 expression in neutrophils in co-culture. Neutrophils were co-cultured with sorted autologous CD4 T cells stimulated with anti-CD3 in the presence or absence of OO, as in Figure 7, but for 16 hours, at a ratio of 1:10 (T: Neutrophils). Following the culture, neutrophils were examined for IL-10 expression. (A) Gating for IL-10 in neutrophils. (B) Comparison of IL-10 expression in neutrophils co-cultured with T cells. Data in blue box were isotype staining controls, and * indicates comparison between IL-10 staining and isotype control. The data in (B) were analyzed by paired Student's *t*-test.
